# Erectile dysfunction among men with chronic kidney disease undergoing hemodialysis in a Brazilian Amazon urban setting: an epidemiological study

**DOI:** 10.1590/2175-8239-JBN-2024-0065en

**Published:** 2024-08-02

**Authors:** João De Barros, Thiago Afonso Teixeira, Fábio Tenorio da Silva, Karina Dias Rocha, Hellen Karine Santos Almeida, Maira Tiyomi Sacata Tongu Nazima

**Affiliations:** 1 Universidade Federal do Amapá, Departamento de Ciências Biológicas e da Saúde, Faculdade de Medicina, Macapá, AP, Brazil.; 2 Universidade Federal do Amapá, Hospital Universitário, Unidade de Urologia, Macapá, AP, Brazil.; 3 Universidade de São Paulo, Instituto de Estudos Avançados, Grupo de Estudos de Saúde do Homem, São Paulo, SP, Brazil.

**Keywords:** Renal Insufficiency, Chronic, Renal Dialysis, Quality of Life, Men’s Health, Sexual Health, Erectile Dysfunction

## Abstract

**Introduction:**

Erectile dysfunction (ED) is a common sexual problem among men with chronic kidney disease (CKD). The severity of sexual dysfunction tends to worsen with kidney damage. This study aims to evaluate the erectile function and sexual quality of life of adult male CKD patients undergoing hemodialysis (HD) in a hospital located in the Brazilian Amazon.

**Methods:**

A cross-sectional quantitative study was performed within the HD Sector of the Nephrology Unit including men with CKD aged ≥ 18 years, undergoing ≥ 3 weekly HD sessions for ≥ 3 months who had been sexually active for ≥ 6 months. We used the Male Sexual Quotient (MSQ) to measure sexual satisfaction and the International Index of Erectile Function (IIEF5) to establish erectile function. Statistical analysis was performed with SPSS 21.0 using appropriate tests, such as Mann-Whitney and Kruskal-Wallis (P < 0.05).

**Results:**

Ninety-eight patients (51.68 ± 15.28 years) were evaluated. They were primarily married/or living with a partner (60.20%), with HD time between 1 to 5 years (55.10%), and an average KTV of 1.17. ED prevalence was 66.30%, and it was associated with a higher age group (p = 0.01), lower family income (p = 0.02), diabetes (p = 0.01), lower mean corpuscular hemoglobin (p = 0.04), higher total calcium (p = 0.04), and lower albumin (p = 0.03). Around 75% classified their sex life as regular to excellent.

**Conclusion:**

Despite the high ED prevalence, most men with CKD in HD reported experiencing regular to excellent sex life. The study underscores the importance of establishing effective screening and conducting routine evaluations regarding sexual issues in these men.

## Introduction

Erectile dysfunction (ED) is a common sexual problem among men with chronic kidney disease (CKD). CKD affects more than 10% of the global population and is a significant global health issue^1^. The severity of sexual dysfunction tends to increase with the level of kidney damage^2^. According to the Brazilian Chronic Dialysis Survey, active dialysis centers increased by 2.7% from 2021 to July 2022, and patients on dialysis increased by 182.1% between 2003 and 2022^3^. In Northern Brazil, there was an 80% increase in the number of patients on dialysis between 2020 and 2022, while in the rest of the country the increase was of 10%^3^.

According to the National Institutes of Health (NIH) consensus, ED is the inability to achieve or maintain an erection sufficient for satisfactory sexual performance^4^. The prevalence of ED can be as high as 80% in individuals with CKD; thus, it is considered a frequent yet potentially treatable complication^5^. ED can lead to a decreased quality of life, causing depression, anxiety, self-esteem issues, and relationship problems. When managing patients with end-stage renal disease, the focus should be on prolonging life expectancy and improving quality of life^6^.

This study aimed to evaluate the erectile function and sexual quality of life of adult male CKD patients undergoing hemodialysis (HD) in a hospital unit in a city in the Brazilian Amazon.

## Methods

We conducted this cross-sectional quantitative study in the Hemodialysis Sector of the Nephrology Unit of the Hospital de Clínicas Dr. Alberto Lima (HCAL) in Macapá, State of Amapá, Brazil. Macapá is a unique Brazilian Amazon city with a distinct blend of indigenous and African cultures and urban lifestyles. Being surrounded by the Amazon River, it is geographically isolated from other developed Amazonian cities and far from the industrialized Brazilian metropolises. Until the end of data collection, this hemodialysis unit provided care for all chronic renal patients on HD in the State of Amapá, so the total number of CKD men receiving HD in the clinic represents this entire specific population for this Brazilian state (N = 105).

We included male patients with CKD aged 18 years or older undergoing three or more weekly hemodialysis sessions for at least three months. Patients who presented clinical instability during the hemodialysis session were excluded from the study. We also excluded indigenous men due to the sensitive sexual issues in this population, which require a culturally appropriate approach under specific Brazilian legislation.

After conducting an epidemiological and clinical survey, two self-administered questionnaires were privately given to individuals who had been sexually active for at least six months: the International Index of Erectile Function (IIEF5) and the Male Sexual Quotient (MSQ). The prevalence of erectile dysfunction was derived from the IIEF5, a self-report questionnaire globally used to classify the quality of erectile function as normal (>21 points), mild (17–21 points), mild to moderate (12–16 points), moderate (8–11 points), and severe (<8 points)^7^. The MSQ is a validated questionnaire designed in Brazil^8^, currently used in various clinical scenarios for Brazilian surveys^9–11^. It was created to measure sexual satisfaction and function across multiple aspects of male sexuality, including desire, confidence, foreplay quality, partner satisfaction, quality of erection, ejaculation control, ability to achieve orgasm, and overall satisfaction with sexual intercourse. It consists of ten self-report questions that use a Likert scale to measure frequency and level of satisfaction. The scores for all ten items were added together and multiplied by 2, resulting in a quotient score on a 100-point scale. We also used the MSQ to identify ejaculatory problems, such as premature ejaculation (PE), delayed ejaculation (DE), and hypoactive sexual desire disorder (HSDD). To estimate the prevalence of each of these sexual dysfunctions, we used the proportion of men with a response score no higher than 2 for each item: HSDD (question 1), “Is your desire strong enough to encourage you to initiate sexual intercourse?” PE (question 8), “Can you control ejaculation so that sexual activity lasts as long as you want?” DE (question 9), “Can you reach orgasm during sex?”. All patients had undergone a medical consultation with the same urologist for clinical confirmation of sexual dysfunctions^8^.

The project was approved by the Research Ethics Committee of the Federal University of Amapá (CAAE 52275516.3.0000.0003), and all patients who participated signed the informed consent form.

We conducted a statistical analysis to determine the measures of central tendency and verify the significance of the differences among groups for quality of sexual life and erectile function. We used approximate 95% confidence intervals. The normality and comparison of samples were assessed using the Kolmogorov-Smirnov test. Non-parametric tests, such as the Mann-Whitney and Kruskal-Wallis tests, were used, depending on the data analyzed. The chi-square test was used to compare non-parametric variables and detect any divergences between observed and expected frequencies. Pearson’s test was employed to determine the degree of linear correlation between two datasets, and the T-test was used to evaluate the differences between group averages. We set the significance level at P < 0.05. All analyses were performed using SPSS 21.0 (IBM Corp, Armonk, NY, USA).

## Results

The population of adult men with CKD consisted of 133 individuals. After applying the exclusion criteria, 105 patients remained, seven of which did not agree to participate. The final sample thus comprised 98 men (average age 51.68 ± 15.28 years). Epidemiologic characteristics are presented in Table 1.

**Table 1:** Baseline demographic parameters of male patients undergoing hemodialysis in Macapá, Brazil

Demographic Parameter		% Patients [N (%)]
Age (years)	<30	7 (7.10%)
	31-40	14 (14.30%)
	41-50	18 (18.40%)
	51-60	35 (35.70%)
	61-70	20 (20.40%)
	71-80	4 (4.10%)
Ethnicity	Brown	57 (58.16%)
	Black	27 (27.60%)
	White	11 (11.22%)
	Yellow	1 (1.02%)
	Others	2 (2.04%)
Marital Status	Married	59 (60.20%)
	Single	29 (29.60%)
	Widower	1 (1%)
	Others	9 (9.20%)
Monthly earned income (US$)	< 317	27 (27.60%)
	317 – 634	16 (16.30%)
	635 – 952	11 (11.20%)
	953 – 1,269	7 (7.10%)
	1,270 – 1,586	5 (5.10%)
	1,587 – 3,174	15 (15.30%)
	> 3,174	17 (17.30%)
Smoking	No smokers	36 (36.73%)
	Ex-smokers	58 (59.18%)
	Active smokers	4 (4.08%)
Tobacco Exposure Time	No smokers	36 (36.73%)
	≤ 20 years	41 (41.83%)
	> 20 years	21 (21.40%)
Alcoholism	No alcoholism	68 (69.38%)
	Ex-alcoholic	16 (16.32%)
	Active alcoholism	14 (14.28%)
Alcoholism Exposure Time	No alcoholism	68 (69.38%)
	≤ 15 years	5 (5.10%)
	> 15 years	25 (25.51%)
Comorbidities	Systemic Arterial Hypertension	87 (88.77%)
	Diabetes Mellitus	38 (38.77%)
	Overweight	37 (37.80%)
	Obesity	17 (17.34%)
Previous Medical History	Acute Myocardial Infarction	10 (10.20%)
	Cerebrovascular accident	3 (3.10%)
Time Undergoing Hemodialysis	3 months to 1 year	16 (16.32%)
	1 year to 5 years	54 (55.10%)
	> 5 years	28 (28.57%)

Out of the total sample, 57 individuals (58,16%) identified as brown. Fifty-nine individuals (60.20%) were either married or in a stable relationship. Thirty-three individuals (33.70%) had no formal education or had studied for up to 8 years. Forty-two individuals (42.90%) were receiving government assistance, 43.90% had a family income of up to US$ 634. Most of the sample (27.60%) had a family income of less than US$ 317.

In the sample group, only 4.08% (n = 4) were active smokers, but the total population with smoking exposure over 20 years was 21.40%. Alcoholics made up 14.28% (n = 14) of the group, while former alcoholics constituted 16.32% (n = 16) with an exposure time of over 15 years, accounting for 25.51% (n = 25) of the sample. The study found that 88.77% of the sample had systemic arterial hypertension (SAH), 38.77% had diabetes mellitus (DM), 17.34% were obese, and 37.80% were overweight. As for previous medical history, 10.20% had experienced a myocardial infarction (MI), and 3.10% had suffered a cerebrovascular accident (CVA). Concerning dialysis treatment time, 55.10% (n = 54) had undergone treatment between 1 and 5 years, while 28.57% (n = 28) had received treatment for more than five years.

The baseline clinical laboratory results are described in Table 2.

**Table 2:** Baseline clinical parameters of male patients undergoing hemodialysis in Macapá, Brazil

Laboratory Parameter	Value (mean ± SD)	Reference Value
Hemoglobin	9.70 ± 2.30 g/dL	14 – 18 g/dL
Hematocrit	28.70 ± 5.81%	38–52%
Pre-dialysis urea	135.00 ± 32.16 mg/dL	16 – 40 mg/dL
Post-dialysis urea	51.51 ± 17.18 mg/dL	16 – 40 mg/dL
Creatinine	11.41 ± 3.17 mg/dL	0.6 – 1.2 mg/dL
Creatinine clearance	5.30 ± 3.30 mL/min/1.73 m^2^	75 – 115 mL/min/1.73 m^2^
Potassium	5.25 ± 0.69 mmol/L	3.5 – 5.5 mmol/L
Calcium	8.30 ± 0.73 mg/dL	8.5 – 10.2 mg/dL
Phosphor	6.43 ± 1.70 mg/dL	2.5 – 4.5 mg/dL
Fasting blood glucose	140.00 ± 81.53 mg/dL	<99 mg/dL
Albumin	3.70 ± 0.34 g/dL	3.5 – 4.7 g/dL
Parathyroid hormone	210.38 ± 241.08 pg/mL	12 – 65 pg/mL
Ferritin	472.48 ± 438.53 ng/mL	23 – 336 ng/mL
Serum iron	72.88 ± 35.42 µg/dL	65 – 175 µg/dL
Transferrin saturation index	29.90 ± 17.00 mg/dL	215 – 365 mg/dL
Alcaline phosphatase	162.38 ± 154.50 U/L	40 – 129 U/L
KTV	1.17 ± 0.22	–

Legends: SD: standard deviation; KTV: dialyzer clearance of urea (K), dialysis time (T), and volume of distribution of urea (V).

### International Index of Erectile Function (IIEF5)

**Figure 1:**
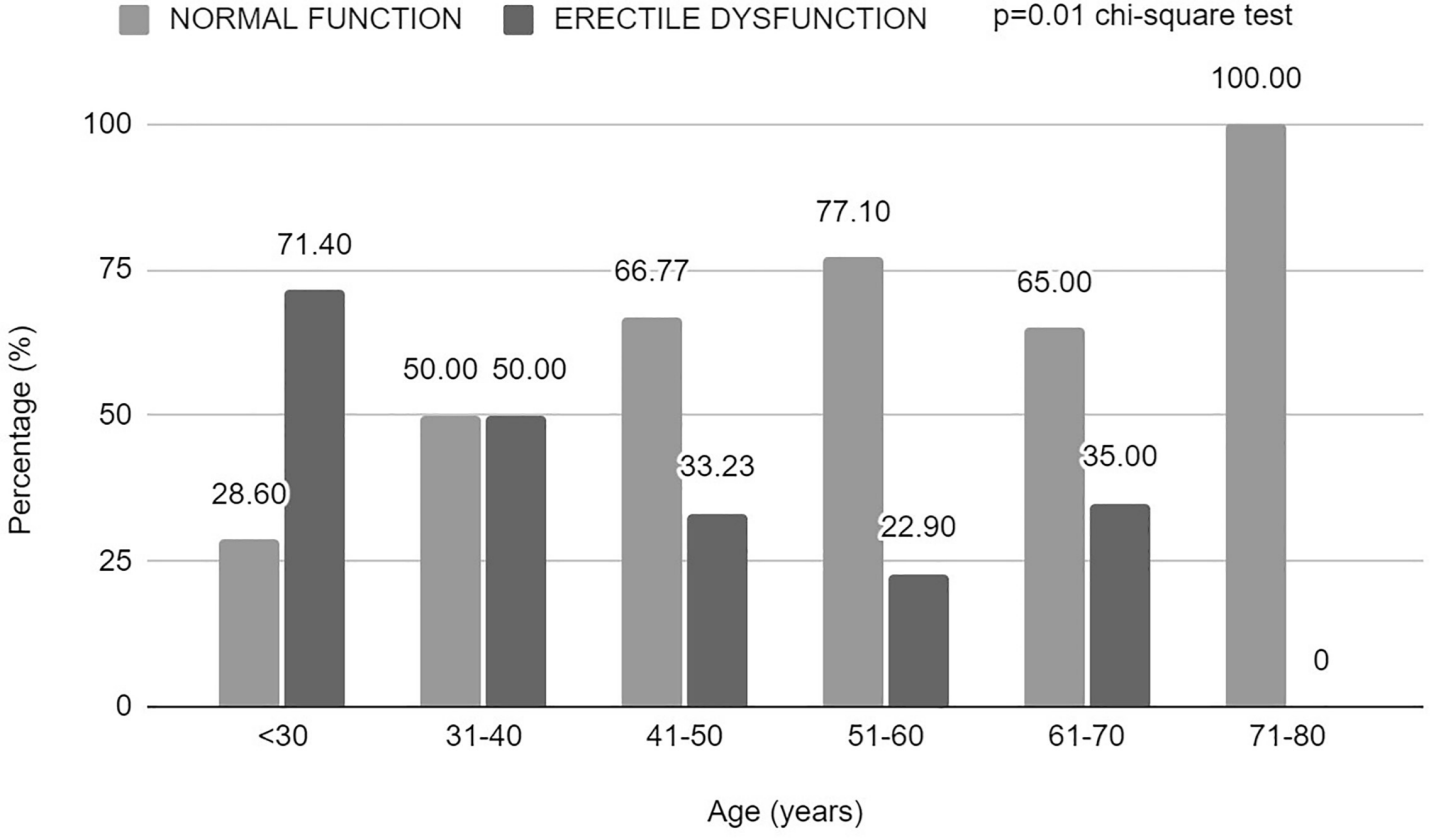
Percentage of men with erectile dysfunction by age group assessed by IIFE5 in male patients undergoing hemodialysis in Macapá, Brazil.

The average value of IIFE5 was 19.45 ± 2.58 (95% CI 18.74–20.17; median of 20), which can be classified as mild erectile dysfunction. ED was reported in 65 cases, 66.30% of the total population. The intensity of ED was distributed as follows: mild (47 cases, 48%), mild to moderate (15 cases, 15.30%), moderate (2 cases, 2%), and severe (1 case, 1%). The prevalence of ED increases with age and reaches a slight stabilization after age 30, only to increase again after age 60 (Figure 1).

**Figure 2:**
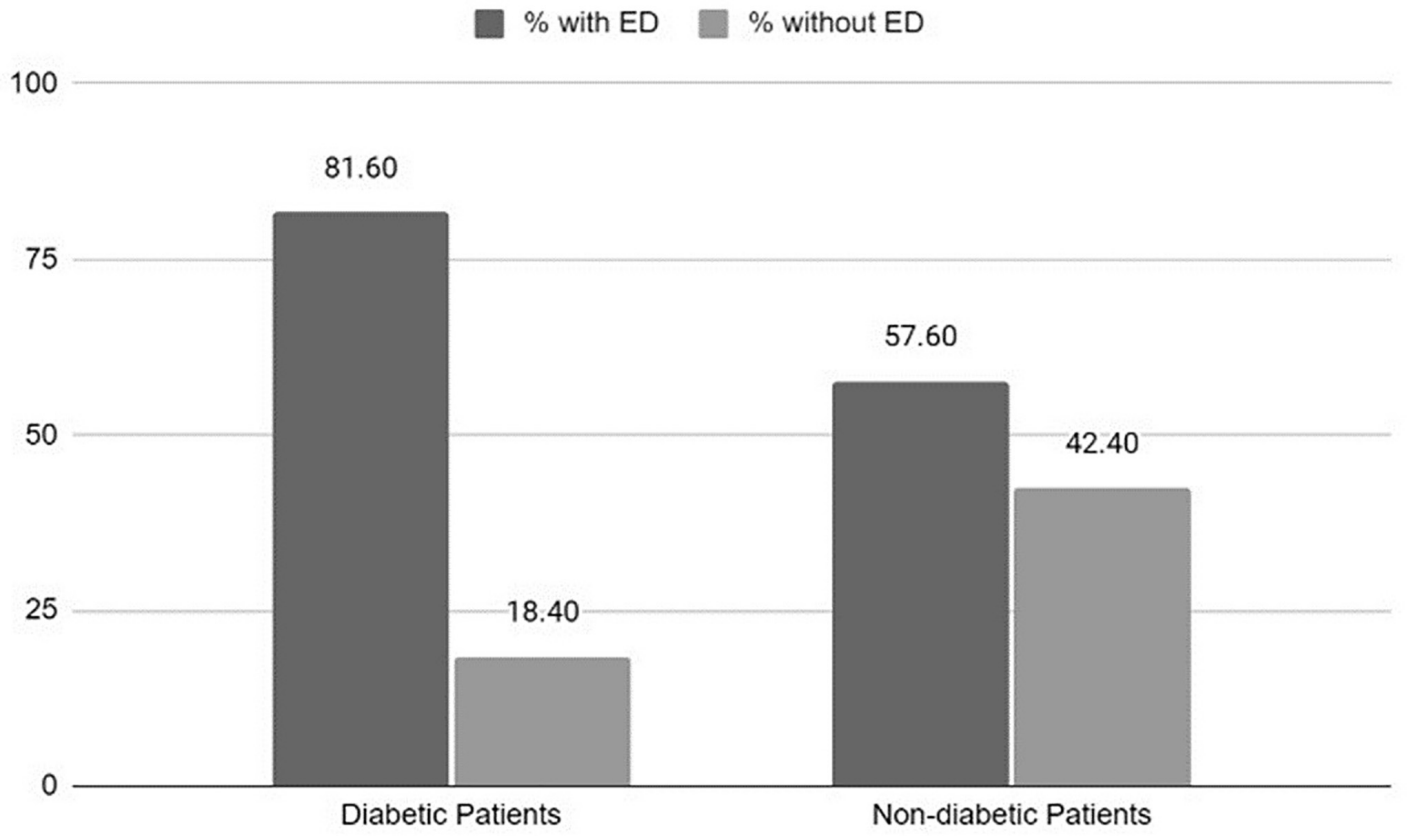
Prevalence of erectile dysfunction according to IIFE in diabetic and non-diabetic patients undergoing hemodialysis in Macapá, Brazil.

Regarding family monthly income, the highest incidence of ED (52.90%) was observed in the group with the highest monthly income (P = 0.02). Among patients with diabetes, 81.60% had ED, while 57.60% of non-diabetic patients had ED (P = 0.01) (Figure 2).

The mean corpuscular hemoglobin (MCH) was lower in subjects with ED compared to subjects with normal function (29.24 vs. 30.18 pg) (P = 0.04), the calcium was higher (8.42 vs. 8.04 mg/dL) (P = 0.04) and the serum albumin was lower (3.69 vs. 3.84 g/dL) (P = 0.03).

### Male Sex Quotient (MSQ)

**Figure 3:**
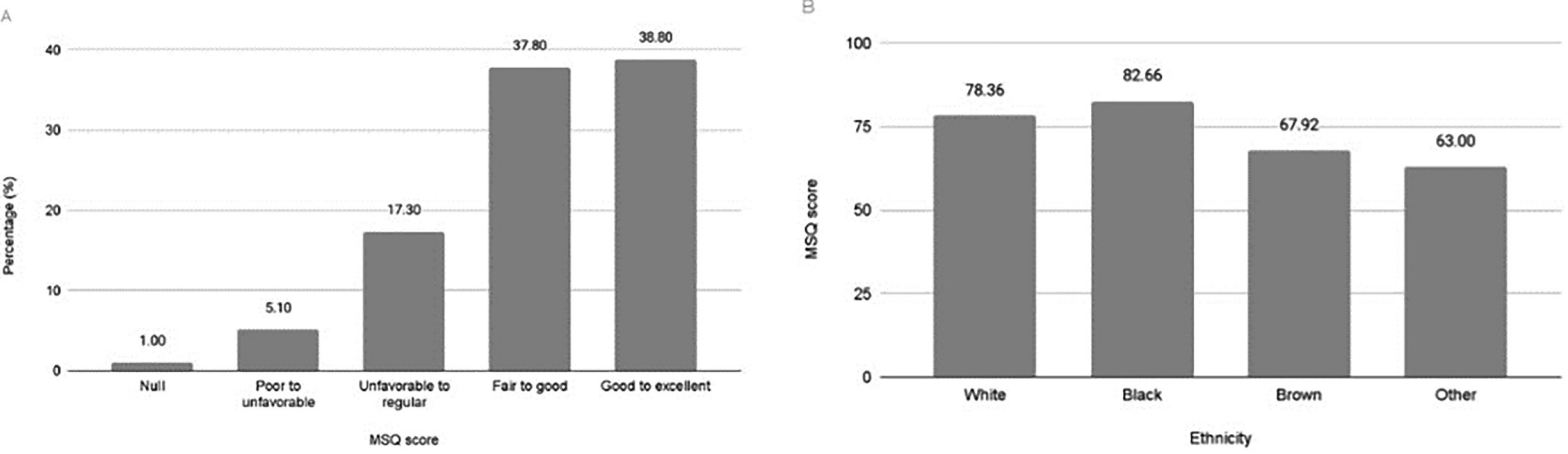
(A) Sexual function classification of male patients undergoing hemodialysis in Macapá according to MSQ. (B) Mean score of individuals according to ethnicity assessed by the MSQ of male patients undergoing hemodialysis in Macapá, Brazil.

In the sample, 1% (n = 1) had a null MSQ score, 5.10% (n = 5) had a poor to unfavorable score, 17.30% (n = 17) had an unfavorable to regular score, 37.80% (n = 37) had a fair to good score, and 38.80% (n = 38) had a good to excellent score. Black individuals scored higher on average (82.66) compared to white (78.36) and brown (67.92) individuals (P = 0.001) (Figure 3).

**Figure 4:**
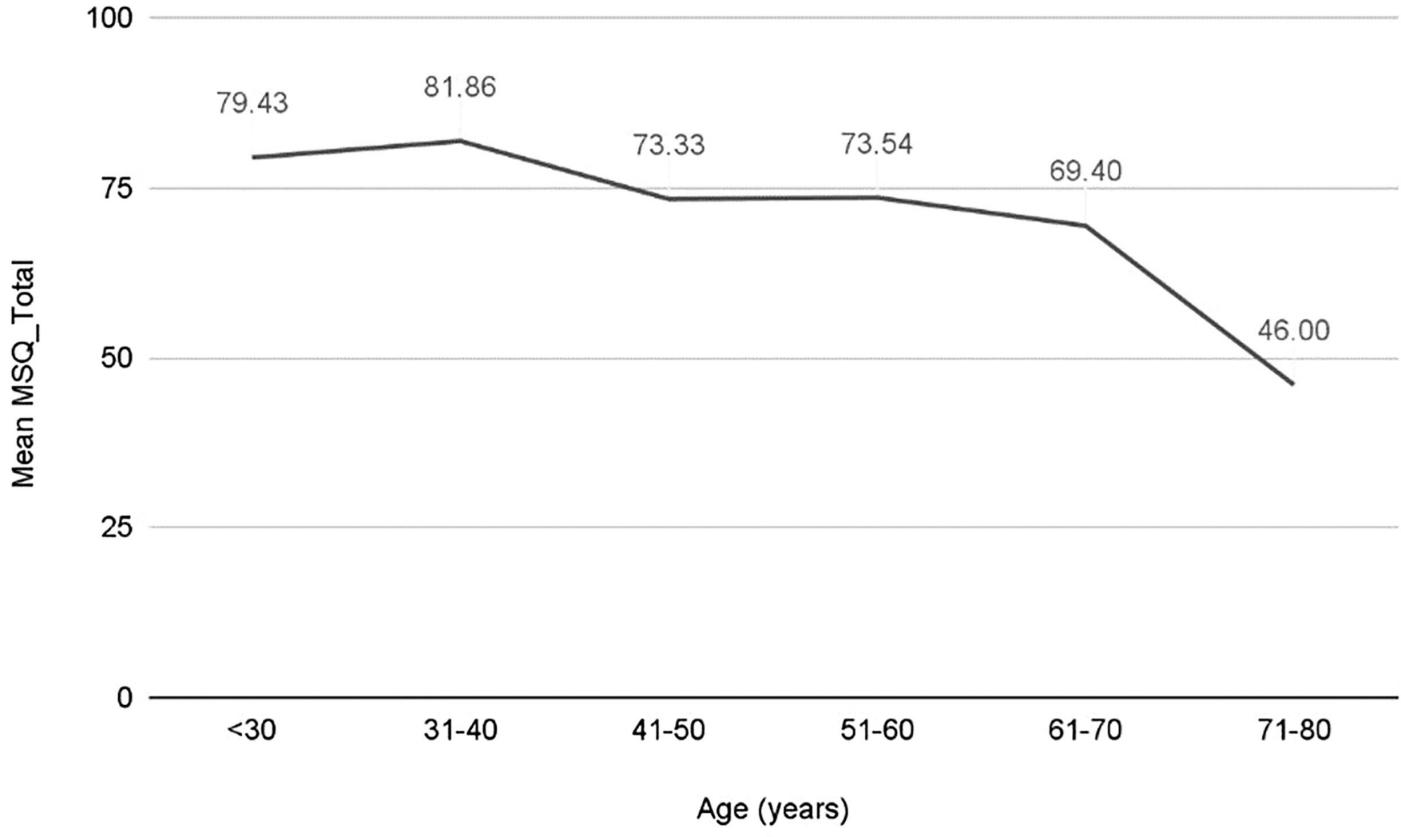
MSQ score according to age groups in patients undergoing hemodialysis in Macapá, Brazil.

The highest scores were observed in patients aged up to 40 (79.43 points for those under 30 and 81.86 for those aged 31–40) (Figure 4). HSDD occurred in 17.30% (n = 17), while PE occurred in 36.70% (n = 36). The DE prevalence was calculated at 14.30% (n = 14).

In comparison to men with CKD and normal erectile function, men with erectile dysfunction had lower mean corpuscular hemoglobin (28.66 vs. 29.83 pg; P = 0.04), lower serum creatinine (9.53 vs. 11.98 mg/dL; P = 0.001), and lower serum albumin (3.59 vs. 3.79 g/dL; P = 0.001), as shown in Table 3.

**Table 3:** Mean corpuscular hemoglobin, serum creatinine, and serum albumin of patients undergoing hemodialysis with and without erectile dysfunction in Macapá, Brazil

Laboratory test	With ED	Without ED
Mean corpuscular hemoglobin	28.66 g/dL	29.83 g/dL
Serum creatinine	9.53 mg/dL	11.98 mg/dL
Serum albumin	3.59 g/dL	3.79 g/dL
Total Serum Calcium	8.42 mg/dL	8.04 mg/dL

Legend: ED: erectile dysfunction.

## Discussion

This research investigated aspects of sexual well-being of men with CKD receiving HD in a specific environment of the world, the Amazon. It highlighted the prevalence of sexual dysfunctions, mainly ED, a clinical concern that is often overlooked in the routine treatment of this patient population.

We reported a higher ED prevalence in adult men with CKD undergoing HD (66.30%), most of which had mild dysfunction (48%). Also, sexual satisfaction was found to be regular to excellent in 76.60%, a value close to that of the local population (80.39%) and the national average (85%)^9,10^.

In men with chronic health conditions, ED is a significant predictor of unsatisfactory sex life^6^. Despite the high rate of unsatisfactory erectile function found in our study, 38.80% of men reported good to excellent sexual satisfaction. It is contradictory if we observe that in a survey by Fugl-Meyer et al.^12^ men with ED complained about their low sexual satisfaction.

Furthermore, Perelman et al.^13^ reported that most men have little or no interest in alternative ways of sexual activity to achieve sexual satisfaction. So, we hypothesized that the local population values other forms of sexual satisfaction, such as a satisfying relationship with a partner and masturbation. Similarly, Corona et al.^14^ state that a normal erection is not essential to be sexually active, and adapting to a more sensory, less coitus-focused sexuality is essential to cope with changes in sexual responses.

As per the literature, this study has also confirmed that patients with CKD have a high incidence of ED^5^, which can be attributed to multiple factors such as reduced arterial blood flow, altered penile muscle function, hormonal imbalances, drug side effects, and neurogenic dysfunction^15^. Rosas et al.^5^ revealed that age, DM (diabetes mellitus), and causes of end-stage renal disease correlate with ED.

CKD can contribute to ED in two ways. Firstly, it can indirectly impact ED through correlated metabolic conditions and secondly, it can directly affect the function of endothelial cells. In patients with CKD, the hypothalamic-pituitary-gonad axis, which is responsible for regulating the reproductive system, can be disrupted, leading to hypogonadism. This condition reduces testosterone production, essential for producing nitric oxide (NO), by upregulating nitric oxide synthase (NOS). NOS is a group of enzymes that catalyze NO production, crucial for achieving and maintaining an erection. Additionally, CKD-induced autonomic neuropathy, which is common in diabetic patients, can also contribute to ED by affecting penile tumescence^16,17^.

As expected, age was directly correlated with ED and lower MSQ scores, and patients with DM had a higher prevalence of ED compared to the non-diabetic group (81.60 vs 57.60%; P = 0.01). Sexual dysfunction affects men of all ages, but studies have shown that it is more prevalent in older men. In our sample, most cases of ED were in the 51-60 age group (27.27%). Fifty percent of patients over 31 have ED, which aligns with the fact that individuals over 40 are more affected^18^.

A cross-sectional observational study found that individuals over 50 were more likely to experience ED^19^. The Massachusetts Male Aging Study found that patients aged 70 years were three times more likely to suffer from severe ED than patients aged 40 years. They also stated that no other variable could explain this result through regression analysis^20^. However, the NIH Consensus Development Panel on Impotence concluded that ED is not caused by age alone, as other risk factors, such as atherosclerosis, may also occur simultaneously with aging^21^.

ED is often an early sign of cardiovascular disease, and one of its most common causes is vascular disorders such as DM, SAH, and dyslipidemia^22^. DM is the primary cause of CKD worldwide and is also considered an independent risk factor for ED in patients with CKD^23^. Up to 75% of men with DM are at risk of developing ED^20,22^. Mesquita et al.^15^ demonstrated that diabetic patients with CKD on conservative treatment had a 4.05 times higher chance of experiencing ED compared to non-diabetic patients. Our study revealed an ED frequency as higher as 80% in men with CKD and DM, significantly higher than CKD patients without DM. We did not find a statistically relevant association between SAH and ED, although SAH is related to ED in patients undergoing HD^23^.

Even though we did not find any correlation between BMI and ED, some studies have suggested a conflicting association between this index and erectile function^24^. While Stolic and Bukumiric found a higher prevalence of ED in obese patients^25^, Molnar et al.^26^ reported more ED cases in underweight patients, particularly men with lower pre-dialysis weight, in a cross-sectional study conducted in Iran. Both malnutrition and obesity have established health implications and are associated with low testosterone levels, which help explain the low erectile function quality found in the literature^24,26^.

While the negative impact of smoking on erectile function is well-known, our research did not find any connection between certain legal drugs and ED. This is consistent with a survey conducted in southern Brazil^27^. However, a study by Costa et al.^19^ found a link between ED, CKD, alcohol consumption, and smoking for more than ten years in patients receiving conservative treatment. Therefore, it is necessary to conduct prospective studies to clarify the correlations between these factors and ED in patients with CKD.

Most patients experienced HD for 1 to 5 years (55.10%). However, there was no significant correlation between HD and ED. This finding is consistent with the study conducted by Arslan et al.^28^. Conversely, Costa et al.^19^ found that time on dialysis was an independent factor for ED. In a survey by Fryckstedt and Hylander^29^, pre-dialysis patients showed less sexual dysfunction than patients on active treatment. However, the degree of dysfunction was not related to treatment modality or medication used. According to a study conducted in Senegal, age and dialysis time were among the most crucial factors for ED^30^.

Our results showed that patients with higher serum creatinine levels had better erectile function outcomes, consistent with other studies that reported a link between ED and lower creatinine levels^31,32^. These results are typical of sarcopenia in elderly patients since creatinine is a marker of muscle mass and tends to be higher in younger patients and in those with no protein-calorie restriction^32^. However, Bodie et al.^33^ suggested that creatinine may increase in men with ED and do not recommend routine screening of serum creatinine.

Like our findings, several studies have found that higher serum albumin levels are linked to better ED outcomes. For instance, a cross-sectional observational study of patients with CKD found that albumin levels below 3.5 g per 100 mL were associated with ED^34^. Similarly, a cross-sectional multicenter study found low serum albumin levels related to ED^35^. Finally, Schiavi et al.^36^ conducted a retrospective and prospective sleep laboratory study and found that albumin-bound testosterone levels correlated positively with erection during sleep in men.

Total calcium was inversely correlated with IIEF-5 score. However, in a cross-sectional study of men and women on peritoneal dialysis, no independent association was found between serum calcium levels and sexual function scores^37^. On the other hand, MCH levels were positively correlated with IIEF-5 and MSQ. Nonetheless, Fugl-Meyer et al.^12^ found a significant decrease in hemoglobin and total testosterone levels associated with increasing stages of CKD.

We found that lower family income was associated with worse erectile function outcomes. Another study supports this result, showing an inverse relationship between the average monthly family income and ED^37^. Interestingly, black individuals obtained the highest MSQ score. However, several studies have failed to prove any association between ethnicity and ED^31^.

PE is a common sexual issue in men with CKD, likely due to abnormalities in serotonergic neurotransmission. Research in animal models suggests that kidney dysfunction affects the regulation of serotonin, controlling ejaculation through receptors in the brain, spinal cord, and peripheral autonomic ganglia^38^. In this study, it was found that 36.70% of the participants suffered from PE. This result was consistent with another study in which a similar PE prevalence (36.54%) was found in the same city but for the general male population^9^. In contrast, DE was reported by 14% of CKD patients, which was more than double the percentage found by Teixeira et al.^9^ (6.50%) for the male adult population of Macapá, Brazil.

Lastly, HSDD was observed in 17.30% of the patients, while the local population had a lower rate of 11.69%^9^. Patients with CKD commonly experience a decrease in libido. This is due to hypogonadism, since sexual hormones play a crucial role in regulating male sexual desire. Additionally, low levels of hemoglobin resulting from reduced erythropoietin in CKD patients seem to be linked to this issue, as treating anemia has shown to improved libido^19^.

According to a study conducted on nephrologists in the Netherlands, sexual problems are not usually discussed in routine check-ups^39^. This is due to factors such as lack of knowledge, inadequate education, and insufficient time, all of which contribute to ED being undervalued in patients with CKD.

It should be noted that there are some limitations to the study, such as the cross-sectional design, which cannot be used to infer causality, and the small sample size, which may have affected the accuracy of some outcomes. Surveys about the prevalence of sexual dysfunction have bias potential, since participants may not report specific sexual issues or might overestimate their performance^40^. Therefore, to minimize these biases, the diagnosis of the sexual health conditions were clinically confirmed by a specialist.

## Conclusion

Finally, this study conducted in Macapá, Brazil, found that 66.30% of adult males with CKD had unsatisfactory erectile function, characterized as ED. Nonetheless, most participants reported regular to excellent sexual quality of life. The study highlights the need for developing effective screening procedures, conducting periodic evaluations, providing sexual psychotherapy, and referring patients to specialists for ED treatment. This is crucial since sexual function plays a significant role in improving the quality of life of men with CKD who are undergoing HD.
