# Supplementary material for: Erectile dysfunction among men with chronic kidney disease undergoing hemodialysis in a Brazilian Amazon urban setting: an epidemiological study
Source: J Bras Nefrol. 2024 Aug 2;46(4):e20240065. doi: 10.1590/2175-8239-JBN-2024-0065en (PMC11309782; doi:10.1590/2175-8239-JBN-2024-0065en)
Supplement: Translation

# Translation: Disfunção erétil entre homens com doença renal crônica em hemodiálise em um cenário urbano da Amazônia brasileira: um estudo epidemiológico

**DOI:** 10.1590/2175-8239-JBN-2024-0065pt

**Authors:** João De Barros, Thiago Afonso Teixeira, Fábio Tenorio da Silva, Karina Dias Rocha, Hellen Karine Santos Almeida, Maira Tiyomi Sacata Tongu Nazima

**Affiliations:** 1 Universidade Federal do Amapá, Departamento de Ciências Biológicas e da Saúde, Faculdade de Medicina, Macapá, AP, Brasil.; 2 Universidade Federal do Amapá, Hospital Universitário, Unidade de Urologia, Macapá, AP, Brasil.; 3 Universidade de São Paulo, Instituto de Estudos Avançados, Grupo de Estudos de Saúde do Homem, São Paulo, SP, Brasil.

**Keywords:** Insuficiência Renal Crônica, Diálise Renal, Qualidade de Vida, Saúde do Homem, Saúde Sexual, Disfunção Erétil

## Abstract

**Introdução:**

Disfunção erétil (DE) é um problema sexual comum entre homens com doença renal crônica (DRC). A gravidade da disfunção sexual tende a piorar com a lesão renal. Este estudo tem como objetivo avaliar função erétil e qualidade de vida sexual de pacientes homens adultos com DRC em hemodiálise (HD) em um hospital na Amazônia brasileira.

**Métodos:**

Realizou-se estudo quantitativo transversal no Setor de HD da Unidade de Nefrologia, incluindo homens com DRC ≥ 18 anos, submetidos a ≥ 3 sessões semanais de HD por ≥ 3 meses e sexualmente ativos por ≥ 6 meses. Utilizamos o Quociente Sexual Masculino (QSM) para medir satisfação sexual e o Índice Internacional de Função Erétil (IIEF5) para estabelecer função erétil. A análise estatística foi realizada com o SPSS 21.0 usando testes apropriados, como Mann-Whitney e Kruskal-Wallis (P < 0,05).

**Resultados:**

Avaliamos 98 pacientes (51,68 ± 15,28 anos). Eles eram predominantemente casados/ou vivendo com um(a) parceiro(a) (60,20%), com tempo de HD entre 1 e 5 anos (55,10%) e KTV médio de 1,17. A prevalência de DE foi 66,30% e foi associada a maior faixa etária (p = 0,01), menor renda familiar (p = 0,02), diabetes (p = 0,01), menor hemoglobina corpuscular média (p = 0,04), maior cálcio total (p = 0,04) e menor albumina (p = 0,03). Aproximadamente 75% classificaram sua vida sexual como regular a excelente.

**Conclusão:**

Apesar da elevada prevalência de DE, a maioria dos homens com DRC em HD relatou vida sexual regular a excelente. O estudo ressalta a importância de estabelecer triagem eficaz e realizar avaliações de rotina sobre questões sexuais nesses homens.

## Introdução

A disfunção erétil (DE) é um problema sexual comum entre homens com doença renal crônica (DRC). A DRC afeta mais de 10% da população mundial e é um problema de saúde global significativo^1^. A gravidade da disfunção sexual tende a aumentar com o nível de lesão renal^2^. De acordo com o Censo Brasileiro de Diálise Crônica, os centros de diálise ativos aumentaram 2,7% de 2021 a julho de 2022 e os pacientes em diálise aumentaram 182,1% entre 2003 e 2022^3^. Na região Norte do Brasil, houve um aumento de 80% no número de pacientes em diálise entre 2020 e 2022, enquanto no restante do país o aumento foi de 10%^3^.

De acordo com o consenso do *National Institutes of Health* (NIH), a DE é a incapacidade de obter ou manter uma ereção suficiente para um desempenho sexual satisfatório^4^. A prevalência de DE pode chegar a 80% em indivíduos com DRC; portanto, é considerada uma complicação frequente, mas potencialmente tratável^5^. A DE pode levar à redução da qualidade de vida, causando depressão, ansiedade, problemas de autoestima e problemas de relacionamento. Ao manejar pacientes com doença renal em estágio terminal, o foco deve ser o prolongamento da expectativa de vida e melhora da qualidade de vida^6^.

O objetivo deste estudo foi avaliar a função erétil e a qualidade de vida sexual de pacientes adultos do sexo masculino com DRC submetidos à hemodiálise (HD) em uma unidade hospitalar de uma cidade da Amazônia brasileira.

## Métodos

Realizamos este estudo quantitativo transversal no Setor de Hemodiálise da Unidade de Nefrologia do Hospital de Clínicas Dr. Alberto Lima (HCAL) em Macapá, Estado do Amapá, Brasil. Macapá é uma cidade amazônica brasileira singular, com uma mistura distinta de culturas indígenas e africanas e estilos de vida urbanos. Por ser cercada pelo rio Amazonas, está geograficamente isolada de outras cidades amazônicas desenvolvidas e distante das metrópoles brasileiras industrializadas. Até o término da coleta de dados, essa unidade de hemodiálise prestava atendimento a todos os pacientes renais crônicos em HD no Estado do Amapá, portanto, o número total de homens com DRC recebendo HD na clínica representa toda essa população específica para esse estado brasileiro (N = 105).

Incluímos pacientes do sexo masculino com DRC com idade igual ou superior a 18 anos, submetidos a três ou mais sessões semanais de hemodiálise por pelo menos três meses. Foram excluídos do estudo os pacientes que apresentaram instabilidade clínica durante a sessão de hemodiálise. Também excluímos os homens indígenas devido às questões sexuais delicadas dessa população, que exigem uma abordagem culturalmente apropriada nos termos da legislação brasileira específica.

Após a realização de uma pesquisa epidemiológica e clínica, dois questionários autoaplicáveis foram entregues de forma privada a indivíduos sexualmente ativos há pelo menos seis meses: o Índice Internacional de Função Erétil (IIEF5, sigla em inglês) e o Quociente Sexual Masculino (QSM). A prevalência da disfunção erétil foi derivada do IIEF5, um questionário de autorrelato utilizado globalmente para classificar a qualidade da função erétil como normal (>21 pontos), leve (17–21 pontos), leve a moderada (12–16 pontos), moderada (8–11 pontos) e grave (<8 pontos)^7^. O QSM é um questionário validado desenvolvido no Brasil^8^ e atualmente utilizado em diversos cenários clínicos para pesquisas brasileiras^9–11^. Foi criado para medir a satisfação e a função sexual em vários aspectos da sexualidade masculina, incluindo desejo, confiança, qualidade das preliminares, satisfação do(a) parceiro(a), qualidade da ereção, controle da ejaculação, capacidade de atingir o orgasmo e satisfação geral com a relação sexual. Ele consiste em dez questões de autorrelato que usam uma escala Likert para medir a frequência e o nível de satisfação. As pontuações para todos os dez itens foram somadas e multiplicadas por 2, resultando em uma pontuação de quociente em uma escala de 100 pontos. Também usamos o QSM para identificar problemas ejaculatórios, como ejaculação precoce (EP), ejaculação retardada (ER) e transtorno do desejo sexual hipoativo (TDSH). Para estimar a prevalência de cada uma dessas disfunções sexuais, utilizamos a proporção de homens com uma pontuação de resposta não superior a 2 para cada item: TDSH (pergunta 1), “Seu desejo é forte o suficiente para incentivá-lo a iniciar a relação sexual?” EP (pergunta 8), “Você consegue controlar a ejaculação para que a atividade sexual dure o tempo que você desejar?” ER (pergunta 9), “Você consegue atingir o orgasmo durante o sexo?”. Todos os pacientes foram submetidos a uma consulta médica com o mesmo urologista para confirmação clínica de disfunções sexuais^8^.

O projeto foi aprovado pelo Comitê de Ética em Pesquisa da Universidade Federal do Amapá (CAAE 52275516.3.0000.0003), e todos os pacientes que participaram assinaram o Termo de Consentimento Livre e Esclarecido.

Realizamos uma análise estatística para determinar as medidas de tendência central e verificar a significância das diferenças entre os grupos quanto à qualidade de vida sexual e à função erétil. Utilizamos intervalos de confiança aproximados de 95%. A normalidade e a comparação de amostras foram avaliadas por meio do teste de Kolmogorov-Smirnov. Foram usados testes não paramétricos, como os testes de Mann-Whitney e Kruskal-Wallis, dependendo dos dados analisados. O teste do qui-quadrado foi utilizado para comparar variáveis não paramétricas e detectar quaisquer divergências entre as frequências observadas e as esperadas. O teste de Pearson foi empregado para determinar o grau de correlação linear entre dois conjuntos de dados, e o teste T foi usado para avaliar as diferenças entre as médias dos grupos. Definimos o nível de significância em P < 0,05. Todas as análises foram realizadas com o SPSS 21.0 (IBM Corp, Armonk, NY, EUA).

## Resultados

A população de homens adultos com DRC foi composta por 133 indivíduos. Após a aplicação dos critérios de exclusão, restaram 105 pacientes, dos quais sete não concordaram em participar. Assim, a amostra final foi composta por 98 homens (idade média de 51,68 ± 15,28 anos). As características epidemiológicas são apresentadas na Tabela 1.

**Tabela 1:** Parâmetros demográficos basais de pacientes do sexo masculino submetidos à hemodiálise em Macapá, Brasil

Parâmetros demográficos		% Pacientes [N (%)]
Idade (anos)	<30	7 (7,10%)
	31–40	14 (14,30%)
	41–50	18 (18,40%)
	51–60	35 (35,70%)
	61–70	20 (20,40%)
	71–80	4 (4,10%)
Etnia	Pardo	57 (58%)
	Preto	27 (27,60%)
	Branco	11 (11,20%)
	Amarelo	1 (1%)
	Outros	2 (2%)
Estado Civil	Casado	59 (60,20%)
	Solteiro	29 (29,60%)
	Viúvo	1 (1%)
	Outros	9 (9,20%)
Renda Mensal (US$)	< 317	27 (27,60%)
	317 – 634	16 (16,30%)
	635 – 952	11 (11,20%)
	953 – 1,269	7 (7,10%)
	1.270 – 1.586	5 (5,10%)
	1.587 – 3.174	15 (15,30%)
	> 3.174	17 (17,30%)
Tabagismo	Não fumantes	36 (36,73%)
	Ex-fumantes	58 (59,18%)
	Fumantes ativos	4 (4,08%)
Tempo de Exposição ao Tabaco	Não fumantes	36 (36,73%)
	≤ 20 anos	41 (41,83%)
	> 20 anos	21 (21,40%)
Etilismo	Não etilista	68 (69,38%)
	Etilistas ativos	14 (14,28%)
	Ex etilistas	16 (16,32%)
Tempo de Exposição ao Etilismo	Não etilista	68 (69,38%)
	≤ 15 anos	5 (5,10%)
	> 15 anos	25 (25,51%)
Comorbidades	Hipertensão arterial sistêmica	87 (88,77%)
	Diabetes Mellitus	38 (38,77%)
	Sobrepeso	37 (37,80%)
	Obesidade	17 (17,34%)
Histórico Médico Anterior	Infarto Agudo do Miocárdio	10 (10,20%)
	Acidente Vascular Encefálico	3 (3,10%)
Tempo de diálise	3 meses a 1 ano	16 (16,32%)
	1 ano a 5 anos	54 (55,10%)
	> 5 anos	28 (28,57%)

Do total da amostra, 57 indivíduos (58,16%) se identificaram como pardos. Cinquenta e nove indivíduos (60,20%) eram casados ou estavam em um relacionamento estável. Trinta e três indivíduos (33,70%) não tinham educação formal ou haviam estudado por até 8 anos. Quarenta e dois indivíduos (42,90%) estavam recebendo assistência governamental, 43,90% apresentavam renda familiar de até US$ 634. A maior parte da amostra (27,60%) apresentava renda familiar inferior a US$ 317.

No grupo amostral, somente 4,08% (n = 4) eram fumantes ativos, mas a população total com exposição ao fumo acima de 20 anos foi de 21,40%. Os alcoólatras representavam 14,28% (n = 14) do grupo, enquanto os ex-alcoólatras constituíam 16,32% (n = 16) com tempo de exposição superior a 15 anos, representando 25,51% (n = 25) da amostra. O estudo constatou que 88,77% da amostra apresentava hipertensão arterial sistêmica (HAS), 38,77% tinham diabetes mellitus (DM), 17,34% eram obesos e 37,80% estavam acima do peso. Quanto ao histórico médico anterior, 10,20% tinham passado por um infarto do miocárdio (IM) e 3,10% haviam sofrido acidente vascular cerebral (AVC). Com relação ao tempo de tratamento de diálise, 55,10% (n = 54) tinham sido submetidos a tratamento entre 1 e 5 anos, enquanto 28,57% (n = 28) haviam recebido tratamento por mais de cinco anos.

Os resultados laboratoriais clínicos basais estão descritos na Tabela 2.

**Tabela 2:** Parâmetros clínicos basais de pacientes do sexo masculino submetidos à hemodiálise em Macapá, Brasil

Parâmetros Laboratoriais	Valor (média ± DP)	Valor de Referência
Hemoglobina	9,70 ± 2,30 g/dL	14 – 18 g/dL
Hematócrito	28,70 ± 5,81%	38–52%
Ureia pré-diálise	135,00 ± 32,16 mg/dL	16 – 40 mg/dL
Ureia pós-diálise	51,51 ± 17,18 mg/dL	16 – 40 mg/dL
Creatinina	11,41 ± 3,17 mg/dL	0,6 – 1,2 mg/dL
*Clearance* de creatinina	5,30 ± 3,30 mL/min/1,73 m^2^	75 – 115 mL/min/1,73 m^2^
Potássio	5,25 ± 0,69 mmol/L	3,5 – 5,5 mmol/L
Cálcio	8,30 ± 0,73 mg/dL	8,5 – 10,2 mg/dL
Fósforo	6,43 ± 1,70 mg/dL	2,5 – 4,5 mg/dL
Glicemia em jejum	140,00 ± 81,53 mg/dL	<99 mg/dL
Albumina	3,70 ± 0,34 g/dL	3,5 – 4,7 g/dL
Paratormônio	210,38 ± 241,08 pg/mL	12 – 65 pg/mL
Ferritina	472,48 ± 438,53 ng/mL	23 – 336 ng/mL
Ferro sérico	72,88 ± 35,42 µg/dL	65 – 175 µg/dL
Índice de saturação de transferrina	29,90 ± 17,00 mg/dL	215 – 365 mg/dL
Fosfatase alcalina	162,38 ± 154,50 U/L	40 – 129 U/L
KTV	1,17 ± 0,22	–

Abreviações: DP: desvio padrão; KTV: depuração de ureia do dialisador (K), tempo de diálise (T) e volume de distribuição da ureia (V).

### Índice Internacional de Função Erétil (IIEF5)

O valor médio do IIFE5 foi de 19,45 ± 2,58 (IC 95% 18,74–20,17; mediana de 20), o que pode ser classificado como disfunção erétil leve. A DE foi relatada em 65 casos, 66,30% da população total. A intensidade da DE foi distribuída da seguinte forma: leve (47 casos, 48%), leve a moderada (15 casos, 15,30%), moderada (2 casos, 2%) e grave (1 caso, 1%). A prevalência de DE aumenta com a idade e atinge uma ligeira estabilização após os 30 anos, apenas para aumentar novamente após os 60 anos (Figura 1).

**Figura 1:** Porcentagem de homens com disfunção erétil por faixa etária avaliados pelo IIFE5 em pacientes do sexo masculino em hemodiálise na cidade de Macapá, Brasil.

Com relação à renda mensal familiar, a incidência mais elevada de DE (52,90%) foi observada no grupo com a maior renda mensal (P = 0,02). Entre pacientes com diabetes, 81,60% apresentaram DE, enquanto 57,60% dos pacientes não diabéticos apresentaram DE (P = 0,01) (Figura 2).

**Figura 2:** Prevalência de disfunção erétil segundo o IIFE em pacientes diabéticos e não diabéticos submetidos à hemodiálise em Macapá, Brasil.

A hemoglobina corpuscular média (HCM) foi menor em indivíduos com DE em comparação com indivíduos com função normal (29,24 vs. 30,18 pg) (P = 0,04), o cálcio foi maior (8,42 vs. 8,04 mg/dL) (P = 0,04) e a albumina sérica foi menor (3,69 vs. 3,84 g/dL) (P = 0,03).

### Quociente Sexual Masculino (QSM)

Na amostra, 1% (n = 1) teve pontuação nula no QSM, 5,10% (n = 5) apresentaram pontuação ruim a desfavorável, 17,30% (n = 17) tiveram pontuação desfavorável a regular, 37,80% (n = 37) tiveram pontuação regular a boa e 38,80% (n = 38) apresentaram pontuação boa a excelente. Os indivíduos negros obtiveram uma pontuação mais elevada em média (82,66) em comparação com indivíduos brancos (78,36) e pardos (67,92) (P = 0,001) (Figura 3).

**Figura 3:** (A) Classificação da função sexual de pacientes do sexo masculino em hemodiálise em Macapá, de acordo com o QSM. (B) Pontuação média dos indivíduos segundo a etnia avaliada pelo QSM de pacientes do sexo masculino em hemodiálise em Macapá, Brasil.

As pontuações mais elevadas foram observadas em pacientes com até 40 anos (79,43 pontos para aqueles com menos de 30 anos e 81,86 para aqueles com 31-40 anos) (Figura 4). O TDSH ocorreu em 17,30% (n = 17), enquanto a EP ocorreu em 36,70% (n = 36). A prevalência de ER foi calculada em 14,30% (n = 14).

**Figura 4:** Pontuação do QSM de acordo com as faixas etárias em pacientes submetidos à hemodiálise em Macapá, Brasil.

Em comparação com homens com DRC e função erétil normal, os homens com disfunção erétil apresentaram hemoglobina corpuscular média mais baixa (28,66 vs. 29,83 pg; P = 0,04), menor creatinina sérica (9,53 vs. 11,98 mg/dL; P=0,001) e menor albumina sérica (3,59 vs. 3,79 g/dL; P = 0,001), conforme mostrado na Tabela 3.

**Tabela 3:** Hemoglobina corpuscular média, creatinina sérica e albumina sérica de pacientes submetidos à hemodiálise com e sem disfunção erétil em Macapá, Brasil

Teste laboratorial	Com DE	Sem DE
Hemoglobina Corpuscular Média	28,66 g/dL	29,83 g/dL
Creatinina Sérica	9,53 mg/dL	11,98 mg/dL
Albumina Sérica	3,59 g/dL	3,79 g/dL
Cálcio Sérico Total	8,42 mg/dL	8,04 mg/dL

Abreviação: DE: disfunção erétil.

## Discussão

Esta pesquisa investigou aspectos do bem-estar sexual de homens com DRC em tratamento de HD em um ambiente específico do mundo, a Amazônia. O estudo destacou a prevalência de disfunções sexuais, principalmente DE, uma preocupação clínica que é frequentemente negligenciada no tratamento de rotina dessa população de pacientes.

Relatamos uma maior prevalência de DE em homens adultos com DRC em HD (66,30%), a maioria apresentando disfunção leve (48%). Além disso, a satisfação sexual foi considerada regular a excelente em 76,60%, um valor próximo ao da população local (80,39%) e da média nacional (85%)^9,10^.

Em homens com condições crônicas de saúde, a DE é um preditor significativo de vida sexual insatisfatória^6^. Apesar da alta taxa de função erétil insatisfatória encontrada em nosso estudo, 38,80% dos homens relataram satisfação sexual boa a excelente. Isso é contraditório se observarmos que, em uma pesquisa realizada por Fugl-Meyer et al.^12^, homens com DE reclamaram de sua baixa satisfação sexual.

Além disso, Perelman et al.^13^ relataram que a maioria dos homens apresenta pouco ou nenhum interesse em formas alternativas de atividade sexual para obter satisfação sexual. Portanto, levantamos a hipótese de que a população local valoriza outras formas de satisfação sexual, como ter um relacionamento satisfatório com o(a) parceiro(a) e a masturbação. Da mesma forma, Corona et al.^14^ afirmam que uma ereção normal não é essencial para ser sexualmente ativo, e a adaptação a uma sexualidade mais sensorial e menos focada no coito é essencial para lidar com as mudanças nas respostas sexuais.

De acordo com a literatura, este estudo também confirmou que pacientes com DRC apresentam uma elevada incidência de DE^5^, que pode ser atribuída a diversos fatores, como fluxo sanguíneo arterial reduzido, função muscular peniana alterada, desequilíbrios hormonais, efeitos colaterais de medicamentos e disfunção neurogênica^15^. Rosas et al.^5^ revelaram que idade, DM (diabetes mellitus) e causas de doença renal em estágio terminal se correlacionam com a DE.

A DRC pode contribuir para a DE de duas maneiras. Primeiramente, pode afetar indiretamente a DE por meio de condições metabólicas correlacionadas e, em segundo lugar, pode afetar diretamente a função das células endoteliais. Em pacientes com DRC, o eixo hipotálamo-hipófise-gônadas, responsável pela regulação do sistema reprodutivo, pode ser interrompido, levando ao hipogonadismo. Esta condição reduz a produção de testosterona, essencial para a produção de óxido nítrico (NO), por meio da regulação positiva da óxido nítrico sintase (NOS). NOS é um grupo de enzimas que catalisam a produção de NO, crucial para atingir e manter uma ereção. Além disso, a neuropatia autonômica induzida pela DRC, que é comum em pacientes diabéticos, também pode contribuir para a DE ao afetar a tumescência peniana^16,17^.

Como esperado, a idade foi diretamente correlacionada com a DE e pontuações mais baixas no QSM, e os pacientes com DM apresentaram maior prevalência de DE em comparação com o grupo não diabético (81,60 vs. 57,60%; P = 0,01). A disfunção sexual afeta homens de todas as idades, mas estudos demonstraram que ela é mais prevalente em homens mais velhos. Em nossa amostra, a maioria dos casos de DE ocorreu na faixa etária de 51 a 60 anos (27,27%). Cinquenta por cento dos pacientes acima de 31 anos têm DE, o que se alinha ao fato de que indivíduos acima dos 40 anos são mais afetados por essa condição^18^.

Um estudo observacional transversal constatou que indivíduos acima de 50 anos apresentaram maior probabilidade de ter DE^19^. O *Massachusetts Male Aging Study* descobriu que pacientes com 70 anos de idade tinham três vezes mais chances de sofrer de DE grave do que pacientes com 40 anos de idade. O estudo também afirmou que nenhuma outra variável poderia explicar esse resultado por meio da análise de regressão^20^. No entanto, o Painel de Desenvolvimento de Consenso sobre Impotência do NIH concluiu que a DE não é causada somente pela idade, já que outros fatores de risco, como a aterosclerose, também podem ocorrer simultaneamente com o envelhecimento^21^.

A DE é frequentemente um sinal precoce de doença cardiovascular e uma de suas causas mais comuns são os distúrbios vasculares, como DM, HAS e dislipidemia^22^. O DM é a principal causa de DRC globalmente e é também considerado um fator de risco independente para DE em pacientes com DRC^23^. Até 75% dos homens com DM correm o risco de desenvolver DE^20,22^. Mesquita et al.^15^ demonstraram que pacientes diabéticos com DRC em tratamento conservador tinham 4,05 vezes mais chances de apresentar DE em comparação com pacientes não diabéticos. Nosso estudo revelou uma frequência de DE de até 80% em homens com DRC e DM, significativamente mais elevada do que em pacientes com DRC sem DM. Não encontramos uma associação estatisticamente relevante entre HAS e DE, embora a HAS esteja relacionada à DE em pacientes submetidos à HD^23^.

Embora não tenhamos encontrado nenhuma correlação entre IMC e DE, alguns estudos sugeriram uma associação conflitante entre esse índice e a função erétil^24^. Enquanto Stolic e Bukumiric encontraram maior prevalência de DE em pacientes obesos^25^, Molnar et al.^26^ relataram mais casos de DE em pacientes abaixo do peso, particularmente homens com menor peso pré-diálise, em um estudo transversal realizado no Irã. Tanto a desnutrição quanto a obesidade têm implicações estabelecidas para a saúde e estão associadas a baixos níveis de testosterona, o que ajuda a explicar a baixa qualidade da função erétil encontrada na literatura^24,26^.

Embora o impacto negativo do tabagismo na função erétil seja bem conhecido, nossa pesquisa não encontrou nenhuma conexão entre certas drogas legais e a DE. Esse fato é consistente com uma pesquisa realizada no sul do Brasil^27^. Entretanto, um estudo realizado por Costa et al.^19^ encontrou uma associação entre DE, DRC, consumo de álcool e tabagismo ao longo de mais de dez anos em pacientes que receberam tratamento conservador. Portanto, é necessário realizar estudos prospectivos para esclarecer as correlações entre esses fatores e a DE em pacientes com DRC.

A maioria dos pacientes foi submetida à HD por 1 a 5 anos (55,10%). Entretanto, não houve correlação significativa entre HD e DE. Esse achado é consistente com o estudo realizado por Arslan et al.^28^. Por outro lado, Costa et al.^19^ constataram que o tempo em diálise foi um fator independente para DE. Em uma pesquisa realizada por Fryckstedt e Hylander^29^, pacientes em pré-diálise apresentaram menos disfunção sexual do que pacientes em tratamento ativo. Entretanto, o grau de disfunção não esteve relacionado à modalidade de tratamento ou ao medicamento utilizado. De acordo com um estudo realizado no Senegal, a idade e o tempo de diálise estavam entre os fatores mais importantes para a DE^30^.

Nossos resultados mostraram que pacientes com níveis mais elevados de creatinina sérica apresentaram melhores desfechos de função erétil, em consonância com outros estudos que relataram uma ligação entre DE e níveis mais baixos de creatinina^31,32^. Esses resultados são típicos de sarcopenia em pacientes idosos, uma vez que a creatinina é um marcador de massa muscular e tende a ser maior em pacientes mais jovens e naqueles sem restrição proteico-calórica^32^. Entretanto, Bodie et al.^33^ sugeriram que a creatinina pode aumentar em homens com DE e não recomendam a triagem de rotina da creatinina sérica.

Assim como nossos achados, diversos estudos constataram que níveis mais elevados de albumina sérica estão associados a melhores desfechos de DE. Por exemplo, um estudo observacional transversal de pacientes com DRC descobriu que níveis de albumina abaixo de 3,5 g por 100 mL estiveram associados à DE^34^. Da mesma forma, um estudo transversal multicêntrico encontrou baixos níveis de albumina sérica relacionados à DE^35^. Por fim, Schiavi et al.^36^ realizaram um estudo retrospectivo e prospectivo em um laboratório do sono e descobriram que os níveis de testosterona ligada à albumina se correlacionavam positivamente com a ereção durante o sono em homens.

O cálcio total foi inversamente correlacionado com o escore IIEF-5. Entretanto, em um estudo transversal de homens e mulheres em diálise peritoneal, não foi encontrada nenhuma associação independente entre os níveis séricos de cálcio e os escores de função sexual^37^. Por outro lado, os níveis de HCM foram positivamente correlacionados com o IIEF-5 e o QSM. No entanto, Fugl-Meyer et al.^12^ encontraram uma redução significativa nos níveis de hemoglobina e testosterona total associada ao aumento dos estágios da DRC.

Descobrimos que a renda familiar mais baixa foi associada a piores desfechos de função erétil. Outro estudo corrobora esse resultado, mostrando uma relação inversa entre a renda familiar média mensal e a DE^37^. Curiosamente, os indivíduos negros obtiveram a pontuação mais elevada no QSM. No entanto, diversos estudos não conseguiram provar qualquer associação entre etnia e DE^31^.

A EP é um problema sexual comum em homens com DRC, provavelmente devido a anormalidades na neurotransmissão serotoninérgica. Pesquisas em modelos animais sugerem que a disfunção renal afeta a regulação de serotonina, controlando a ejaculação por meio de receptores no cérebro, medula espinhal e gânglios autonômicos periféricos^38^. Neste estudo, constatou-se que 36,70% dos participantes sofriam de EP. Esse resultado foi consistente com outro estudo no qual foi encontrada uma prevalência semelhante de EP (36,54%) na mesma cidade, mas para a população masculina em geral^9^. Em contraste, a ER foi relatada por 14% dos pacientes com DRC, mais que o dobro da porcentagem encontrada por Teixeira et al.^9^ (6,50%) para a população adulta masculina de Macapá, Brasil.

Por fim, o TDSH foi observado em 17,30% dos pacientes, enquanto a população local apresentou uma taxa menor, de 11,69%^9^. Os pacientes com DRC geralmente apresentam uma redução da libido. Isso se deve ao hipogonadismo, uma vez que os hormônios sexuais desempenham um papel crucial na regulação do desejo sexual masculino. Além disso, baixos níveis de hemoglobina resultantes da redução da eritropoietina em pacientes com DRC parecem estar ligados a esse problema, já que o tratamento da anemia demonstrou melhorar a libido^19^.

De acordo com um estudo realizado com nefrologistas na Holanda, os problemas sexuais geralmente não são discutidos em exames de rotina^39^. Isso se deve a fatores como falta de conhecimento, educação inadequada e tempo insuficiente, os quais contribuem para que a DE seja subvalorizada em pacientes com DRC.

Deve-se observar que existem algumas limitações no estudo, como o desenho transversal, que não pode ser utilizado para inferir causalidade, e o pequeno tamanho da amostra, que pode ter afetado a precisão de alguns desfechos. Pesquisas sobre a prevalência de disfunção sexual têm potencial de viés, uma vez que os participantes podem não relatar problemas sexuais específicos ou podem superestimar seu desempenho^40^. Portanto, para minimizar esses vieses, o diagnóstico das condições de saúde sexual foi confirmado clinicamente por um especialista.

## Conclusão

Por fim, esse estudo realizado em Macapá, Brasil, constatou que 66,30% dos homens adultos com DRC apresentaram função erétil insatisfatória, caracterizada como DE. No entanto, a maioria dos participantes relatou qualidade de vida sexual regular a excelente. O estudo destaca a necessidade de desenvolver procedimentos de triagem eficazes, realizar avaliações periódicas, fornecer psicoterapia sexual e encaminhar os pacientes a especialistas para tratamento da DE. Isso é fundamental, uma vez que a função sexual desempenha um papel significativo na melhoria da qualidade de vida dos homens com DRC que estão em tratamento de HD.
